# Associations among Dietary Omega-3 Polyunsaturated Fatty Acids, the Gut Microbiota, and Intestinal Immunity

**DOI:** 10.1155/2021/8879227

**Published:** 2021-01-02

**Authors:** Yawei Fu, Yadong Wang, Hu Gao, DongHua Li, RuiRui Jiang, Lingrui Ge, Chao Tong, Kang Xu

**Affiliations:** ^1^College of Animal Science and Technology, Henan Agricultural University, Zhengzhou, Henan, China; ^2^Key Laboratory of Agro-ecological Processes in Subtropical Region, Institute of Subtropical Agriculture, Chinese Academy of Sciences, Changsha, Hunan, China; ^3^Hunan Biological and Electromechanical Polytechnic, Changsha, Hunan, China; ^4^State Key Laboratory of Developmental Biology of Freshwater Fish, College of Life Sciences, Hunan Normal University, Changsha, Hunan, China

## Abstract

Omega-3 polyunsaturated fatty acids (omega-3 PUFAs), which are essential fatty acids that humans should obtain from diet, have potential benefits for human health. In addition to altering the structure and function of cell membranes, omega-3 PUFAs (docosahexaenoic acid (DHA), eicosapentaenoic acid (EPA), alpha-linolenic acid (ALA), and docosapentaenoic acid (DPA)) exert different effects on intestinal immune tolerance and gut microbiota maintenance. Firstly, we review the effect of omega-3 PUFAs on gut microbiota. And the effects of omega-3 PUFAs on intestinal immunity and inflammation were described. Furthermore, the important roles of omega-3 PUFAs in maintaining the balance between gut immunity and the gut microbiota were discussed. Additional factors, such as obesity and diseases (NAFLD, gastrointestinal malignancies or cancer, bacterial and viral infections), which are associated with variability in omega-3 PUFA metabolism, can influence omega-3 PUFAs–microbiome–immune system interactions in the intestinal tract and also play roles in regulating gut immunity. This review identifies several pathways by which the microbiota modulates the gut immune system through omega-3 PUFAs. Omega-3 supplementation can be targeted to specific pathways to prevent and alleviate intestinal diseases, which may help researchers identify innovative diagnostic methods.

## 1. Introduction

Gut microbes play vital roles in maintaining intestinal health [1]. Nutrients exert profound effects on gut microbes and intestinal immunity. Nutrients and intestinal immunity are mediated by gut microbes, and there is a strong correlation between these factors. Omega-3 PUFAs, particularly DHA, are widely used, as they promote the intellectual development of children [2]. As essential fatty acids, dietary omega-3 PUFAs participated in regulating gut immunity and the maintenance of gut homeostasis, which are associated with the gut microbiota, fatty acid metabolism, and intestinal health [3]. In this review, we discuss how omega-3 PUFAs interact with the gut microbiota, how omega-3 PUFAs modulate gut immunity, and the relationship between gut microbes and intestinal immunity. The factors that alter the interaction among omega-3 PUFAs, gut microbes, and intestinal immunity will be discussed. These discussions might provide new insights into the prevention or treatment of diseases related to disorders of omega-3 PUFA metabolism or intestinal microbes.

## 2. Omega-3 Polyunsaturated Fatty Acids

**Figure 1:**
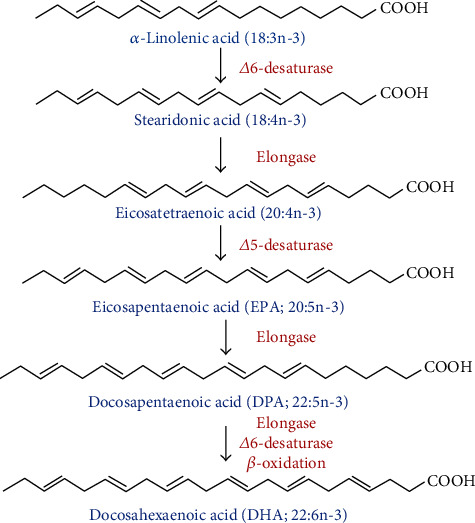
Synthetic pathway of omega-3 polyunsaturated fatty acids.

Omega-3 PUFAs including EPA, DHA, and ALA are essential fatty acids for animals [4]. DPA is an intermediate between EPA and DHA. EPA and DHA are mainly derived from marine organisms or deep-sea fish, such as salmon, sardines, and mackerel [5]. Omega-3 PUFAs cannot be synthesized by the human body and must be directly supplied by diet or converted from ingested ALA. Only a small fraction of ALA can be converted to EPA, DPA, or DHA (Figure 1), so dietary supplements or pharmaceutical preparations are essential to provide sufficient unsaturated fatty acids [6]. In addition to being an energy source for body, omega-3 PUFAs play important roles in infant brain development and relieving inflammation [7]. The addition of omega-3 PUFAs to the diet could decrease LDL-cholesterol, prevent myocardial infarction, and reduce the morbidity and mortality of cardiovascular disease [8–10]. Omega-3 PUFAs are widely ingested through food or supplements, which was considered to exert additional beneficial effects throughout the whole body, so the effect of omega-3 PUFAs on gut microbes is a topic worth exploring. The abundance of human gut microbes is positively correlated with the concentration of omega-3 PUFAs in the blood [11]. Currently, omega-3 PUFAs have become one of the hotspots in nutritional biochemistry research and play important roles in regulating gut microbes and gut immunity [12].

## 3. Omega-3 PUFAs and the Gut Microbiota

Accumulating evidence implicates the correlation between omega-3 PUFAs and gut microbiota. Omega-3 PUFAs can influence the gut microbial community; in turn, gut microbiota can also affect the metabolism and absorption of omega-3 PUFAs. However, the knowledge about the connections between omega-3 PUFAs and gut microbiota is limited. In adults, changes in the gut microbiota were observed after omega-3 PUFA supplementation [3]. Coincidentally, changes in gut microbiota were observed in patients with intestinal inflammation. The connections between omega-3 PUFAs and gut microbiota will be discussed in the following sections.

### 3.1. Omega-3 PUFAs Could Affect Gut Microbiota

Omega-3 PUFAs affect the gut microbiome in three main ways: (1) modulating the type and abundance of gut microbes; (2) altering the levels of proinflammatory mediators, such as endotoxins (lipopolysaccharides) and IL17; and (3) regulating the levels of short-chain fatty acids or short-chain fatty acid salts.

Omega-3 PUFAs could directly modulate the diversity and abundance of the gut microbiota. Compared with sunflower oil, the dietary intake of fish oil exerted the greatest effect on the diversity of the intestinal flora [13]. High levels of omega-3 PUFAs in fish oil cause significant changes in the gut microbiota, which might explain the health benefits of its use [14]. In addition, fish oil exerts an inhibitory effect on a variety of bacteria. Omega-3 PUFAs could exert beneficial effects on the gut microbiota through decreasing the growth of *Enterobacteria*, increasing the growth of *Bifidobacteria*, and subsequently inhibiting the inflammatory response associated with metabolic endotoxemia [15].

Studies using animal models show the association between fatty acid ingestion and changes in gut microbiota [16]. Omega-3 PUFAs, obtained from the diet, are partially metabolized by anaerobic bacteria, such as *Bifidobacteria* and *Lactobacilli*, in the distal intestine, thus affecting the distribution of the intestinal flora [17]. In addition, omega-3 PUFAs can also increase the number and abundance of beneficial bacteria, such as *Bifidobacterium* [18]. Dietary addition of omega-3 PUFAs increases the abundance and percentage of *Bifidobacteria* in the gut of male Sprague-Dawley rats [19]. EPA and DHA treatment could prevent gut microbiota dysregulation in mice [20] and increase the number of potentially beneficial lactic acid-producing bacteria and *Bifidobacteria* in the gut of the mice fed a high-fat diet [21, 22]. Omega-3 PUFAs alter the abundance of beneficial intestinal bacteria, particularly *Akkermansia*, improve the intestinal microenvironment, increase the intestinal mucosal thickness, improve the barrier function of the intestinal mucosa, and achieve weight loss by controlling the expression of genes related to fat metabolism [23].

Omega-3 PUFAs could directly or indirectly alter the balance of gut microbes, contributing to the occurrence and development of multiple diseases [24]. Omega-3 PUFAs modulate the content of the gut microbiota [25, 26]. Firmicutes and Bacteroidetes are two major bacterial phyla that dominate the human gut microbiota. The Firmicutes-to-Bacteroidetes ratio (F/B ratio) is associated with obesity, nonalcoholic fatty liver disease (NAFLD), and other diseases. An imbalanced intake of omega-3/omega-6 PUFAs may lead to gut microbe dysbiosis, particularly a significant increase of the F/B ratio, which eventually leads to overweight and obesity [27]. Dietary omega-3 PUFAs are able to attenuate the decrease of the F/B ratio observed in high-fat diet-fed mice [28]. Furthermore, omega-3 PUFAs could improve the condition of patients with IBD by reverting the microbiota to a healthier composition [29]. The increase of the abundance of the *Escherichia*, *Faecalibacterium*, *Streptococcus*, *Sutterella*, and *Veillonella* genera and the decrease of the abundance of the *Bacteroides*, *Flavobacterium*, and *Oscillospira* genera were detected in the IBD group after supplementation with omega-3 PUFAs, presenting the decreased F/B ratio [30]. Furthermore, omega-3 PUFA supplementation may attenuate early life stress-induced perturbations in the gut microbiota [31]. In summary, omega-3 PUFAs directly alter the diversity and abundance of gut microbes, particularly the F/B ratio.

Omega-3 PUFAs also can modulate gut microbes through inhibiting the production of proinflammatory mediators or promoting the production of anti-inflammatory mediators. In some cases, LPS passes through the intestinal wall, particularly when the barrier is destroyed, causing further damage [32]. The increased intestinal permeability will in turn result in the accumulation of toxic bacterial products such as LPS and bacterial DNA in the hepatic portal circulation [33]. Even small amounts of LPS in the systemic circulation, measured in picogram, have the potential to cause an inflammatory response in humans [34].

The consumption of omega-3 PUFAs inhibits the LPS-induced production of proinflammatory cytokines in human blood monocytes [35], relieves intestinal inflammation, and maintains a steady state of gut microbes. Omega-3 PUFAs inhibit all NF-*κ*B pathways induced by LPS. Incubation of macrophages with omega-3 PUFAs reduces MAPK kinase activity induced by LPS and decreases the expression of proinflammatory mediators, such as TNF-*α* [36]. Omega-3 PUFAs promote the release of large amounts of anti-inflammatory factors such as IL-10 from resident macrophages, promote the induction of regulatory T cells (Tregs), and prevent the overdevelopment of T helper 17 (Th17) cells [37]. Interleukin 17 (IL-17), a proinflammatory cytokine produced primarily by Th17 cells, causes tissue inflammation. Omega-3 PUFAs may reduce gut inflammation by increasing Treg differentiation and decreasing IL-17 production [38].

Omega-3 PUFAs can also affect gut microbes through increasing the content of SCFAs. Omega-3 PUFAs exert a positive effect through restoring the microbiota composition in individuals with various diseases and increasing the production of anti-inflammatory compounds, such as SCFAs [39]. Butyric acid-producing bacteria play an important role in maintaining human gut health by degrading nonfermentable dietary fibers into SCFAs, such as butyrate [40]. Butyrate is considered an essential energy source for the colonic mucosa that controls gene expression, inflammation, differentiation, and apoptosis in host cells [41]. The addition of omega-3 PUFAs to *Salmonella*-infected mice significantly increased the SCFA content, thereby altering the gut microbiota and favoring host resistance to pathogens [42]. In one case report of the effect of an omega-3 PUFA-rich diet on human intestinal microbiota, a significant increase in several SCFA (butyrate)-producing genera, including *Blautia*, *Bacterioides*, *Roseburia*, and *Coprococcus*, was observed [43]. Increased daily intake of 4 g of mixed omega-3 PUFAs (DHA and EPA) significantly increased the density of bacteria that are known to produce butyrate. Butyrate-producing bacteria play a key role in maintaining human gut health by degrading nonfermentable dietary transfer into short-chain fatty acids (SCFAs), such as butyrate [44, 45].

The effect of omega-3 PUFAs on the gut microbiota may be a main contributor to the health benefits of omega-3 PUFAs. Omega-3 PUFAs are mainly absorbed in the gut, where some microorganisms can directly utilize omega-3 PUFAs and produce numerous small molecules. Studies have highlighted the changes in the gut microbiota after omega-3 PUFAs supplementation [22]. Further studies should provide additional insights into the associations among the gut microbiota, omega-3 PUFAs, and intestine health [46].

### 3.2. Effect of Intestinal Microbes on the Metabolism and Absorption of Omega-3 PUFAs

Omega-3 PUFAs could directly affect the gut microbiota, and correspondingly, the gut microbiota could directly or indirectly modulate the absorption, bioavailability, and biotransformation of omega-3 PUFAs and further influence the imbalance of PUFA intake and its function. Gut microbes produce PUFA-derived metabolites, which may be novel active metabolites [47]. As shown in animal models, microorganisms play an essential role in the biotransformation of PUFAs. Some microbial species, such as *Bacillus proteus* or *Lactobacillus plantarum*, convert the omega-3/omega-6 PUFA precursors ALA and LA into CLA (conjugated linoleic acids) and CALA (conjugated *α*-linolenic acids), respectively, which are then further hydrogenated to saturated fatty acids (stearic acid, C18:0), thereby reducing PUFA composition [48]. PUFA-derived intermediate metabolites are produced by a wide range of bacteria, including lactic acid-producing bacteria. In addition, the in vitro stimulation and in vivo administration of PUFA-derived bacterial metabolites results in antiobesity and anti-inflammatory effects [49].

The intestinal flora affects host health or nutrition-related diseases through regulating the digestion and absorption of PUFAs [50]. The main source of omega-3 PUFAs is the diet, and some microorganisms in the intestine directly alter the availability of omega-3 PUFAs. *Bifidobacterium* modulates fatty acid metabolism or fatty acid uptake by the intestinal epithelium, but the mechanism underlying the association between *Bifidobacterium* and the absorption of omega-3 PUFAs was not elucidated [51]. Interactively, dietary intake of omega-3 PUFAs may increase the abundance of *Bifidobacterium* in the gut. An increase in the relative abundance of *Bifidobacterium* in the gut via the administration of probiotics or prebiotics also increases the blood omega-3 PUFA levels, which is beneficial to our health, such as preventive and therapeutic effects on cardiovascular diseases and affective disorders.

The effects of gut microbes on the metabolism and absorption of omega-3 PUFAs may be mediated by SCFAs. Omega-3 PUFA supplementation induces a reversible increase in the abundance of several SCFA-producing bacteria, containing *Bifidobacterium*, *Roseburia*, and *Lactobacillus* in the mouse intestinal tract [52]. In mice, high levels of omega-3 PUFAs in tissue are associated with differences in intestinal microbiota, such as *Bifidobacterium* and *Lactobacillus* [53]. Based on studies, *Bifidobacterium* may be the main genus of bacteria that modulates the utilization of omega-3 PUFAs by microorganisms. Further studies are needed to explore the relationship between *Bifidobacterium* and omega-3 PUFAs.

## 4. Omega-3 PUFAs and Inflammation

### 4.1. Omega-3 PUFAs Affect Intestinal Immunity

Omega-3 PUFAs can improve intestinal immunity. Omega-3 PUFAs could reduce intestinal epithelial cell damage caused by LPS, sodium dextran sulfate, or hydrogen peroxide and increase intracellular mitochondrial activity and cell membrane integrity [54]. Stress exposure increases intestinal dysfunction and decreases intestinal immunity. Chronic stress causes a series of anomalies in the intestine, including a decreased fecal water content, increased production of proinflammatory cytokines (TNF-*α*, IL-1*β*, IFN-*γ*, and IL-6), and aberrant changes in the microbiota composition (particularly *Bifidobacterium*, *Lactobacillus*, and *Roseburia* and *Prevotella* spp.). Omega-3 PUFAs have been shown to effectively counteract these adverse effects [15].

Omega-3 PUFAs modulate intestinal immunity through three main mechanisms. First, omega-3 PUFAs reduce the release of membrane phospholipid arachidonic acid (AA) by reducing the intracellular AA content or by inhibiting phospholipase activity [55]. Second, omega-3 PUFAs inhibit NF-*κ*B-mediated inflammation or attenuate the phosphorylation of MAPKs, subsequently reducing the transcription of inflammatory molecules [56]. Finally, the intake of omega-3 PUFA modifies the gut microbiome and ameliorates dysbiosis by increasing the abundance of lactic acid-producing bacterial species and reducing the abundance of *Bacillus* species. The ingestion of omega-3 PUFAs inhibits LPS-induced proinflammatory cytokine production in human blood monocytes [57]. Omega-3 PUFAs modulate intestinal immunity in many ways, and the studies described above have provided some avenues and evidence, but further studies are needed.

### 4.2. Omega-3 PUFAs on Inflammation

Accumulating evidences revealed omega-3 PUFAs, primarily EPA and DHA, suppress inflammation and exert a beneficial effect on a variety of inflammation-related diseases, such as inflammatory bowel disease, rheumatoid arthritis, asthma, cancer, and cardiovascular diseases [58]. PUFAs suppress immune responses and are used as adjuvant immunosuppressive agents in the clinic to treat inflammatory diseases (rheumatoid arthritis and IBD) or after organ transplantation [59]. Omega-3 PUFAs are known to interfere with the synthesis of proinflammatory eicosanoids [22]. However, PUFA-mediated inhibition of T lymphocyte activation and function has been repeatedly shown to be independent of eicosanoid synthesis.

**Figure 2:**
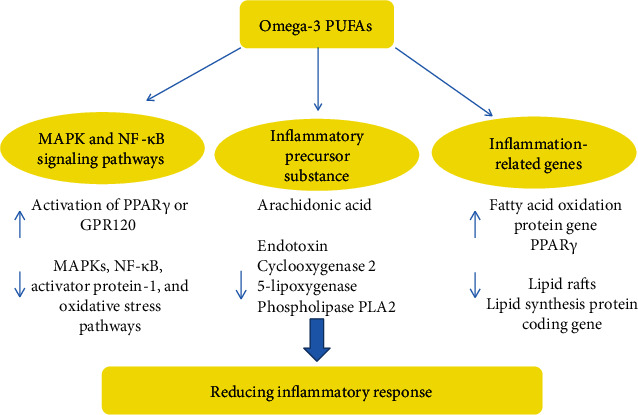
Omega-3 PUFAs reduce inflammation through three main pathways.

Omega-3 PUFAs may reduce inflammation through three main pathways: (1) mediating immune cell activation through the MAPK and NF-*κ*B signaling pathways, (2) reducing the production of precursors that cause inflammation, and (3) altering the mechanism regulating the expression of inflammation-related genes (Figure 2).

Omega-3 PUFAs reduce inflammation by decreasing the activation of proinflammatory MAPK, NF-*κ*B, activator protein-1, and oxidative stress pathways or through increasing the activation of PPAR*γ* or GPR120. Given the proinflammatory effects of several MAPKs, particularly extracellular signal-related kinases and c-Jun N-terminal kinase (JNK), the inhibition of specific MAPKs is a prospective mechanism by which omega-3 PUFAs block or reduce intestinal inflammation. Omega-3 PUFAs maintain intestinal health by reducing oxidative stress and NF-*κ*B-mediated inflammation in immune cells and intestinal cells [60]. Omega-3 PUFAs inhibit NF-*κ*B signaling by activating peroxisome proliferator-activated receptor (PPAR)-*γ* [61, 62].

Another possible mechanism is the suppression of inflammation through the activation of GPR120, an omega-3 fatty acid-activated receptor expressed in white adipose tissue (WAT) and bone marrow-derived dendritic cells and macrophages [63, 64]. For example, in the Sprague-Dawley rat model, supplementation with an equal mixture of EPA and DHA reduced intestinal barrier dysfunction and reversed the decrease in PPAR-*γ* levels in the intestine due to ischemia and reperfusion injury [15]. Thus, the amount of evidence has confirmed the anti-inflammatory effects of supplementation with long-chain omega-3 PUFAs. Among the components of a healthy diet, the intake of omega-3 fatty acids is associated with reduced inflammation [65].

The consumption of a diet rich in omega-3 PUFAs has been reported to protect intestinal cells from inflammatory damage that leads to IBD and to activate immune cells by reducing the production of proinflammatory eicosanoids. Omega-3 PUFAs may also exert their anti-inflammatory effects through incorporation into the plasma or phospholipid membranes of immune cells or intestinal mucosal tissues in human and rodent models [66]. Furthermore, studies using omega-3 desaturase transgenic mice enriched in endogenous omega-3 PUFAs strongly support the hypothesis that omega-3 PUFAs exert a protective effect on inflammatory pathology [67]. Omega-3 PUFAs serve as alternative substrates for cyclooxygenase (COX) or lipoxygenase (LOX), preventing the conversion of arachidonic acid (AA) to the proinflammatory eicosanoid and reducing the production of inflammatory factors [68]. In summary, omega-3 PUFAs reduce inflammation by incorporating into phospholipid membranes, where they inhibit the production of proinflammatory eicosanoids and reduce the immune cell activation and the release of proinflammatory cytokines [69, 70].

Some of the beneficial effects of PUFAs are attributed to changes in the fatty acid composition of the membrane and subsequent alterations in hormone signaling. Omega-3 PUFAs disrupt lipid rafts and inhibit the activation of the proinflammatory transcription factor NF-*κ*B, thereby reducing the expression of inflammatory genes and activating the anti-inflammatory transcription factor PPAR*γ* [71]. The fatty acids themselves exert direct, membrane-independent effects on the molecular events that control gene expression. The regulation of gene expression by dietary fat exerts the greatest effect on the development of insulin resistance and its associated pathophysiology. PUFAs exert their beneficial effects by upregulating the expression of genes involved in fatty acid oxidation while downregulating genes encoding proteins involved in lipid synthesis [72]. PUFAs regulate the expression of oxidative stress-related genes by activating the transcription factor peroxisome proliferator-activated receptor. PUFAs inhibit the expression of lipogenic genes by decreasing the nuclear abundance and DNA-binding affinity of transcription factors responsible for inducing the expression of lipogenic and glycolytic genes to control insulin and carbohydrate levels [73, 74].

Omega-3 PUFAs also alleviate alcoholic steatosis and alcohol-induced liver injury through various mechanisms, including reducing adipose tissue lipogenesis and lipid mobilization, enhancing mitochondrial fatty acid *β*-oxidation, reducing hepatic inflammation and oxidative stress, and promoting intestinal homeostasis, suggesting that omega-3 PUFAs may be promising treatments in the management of alcoholic liver disease (ALD) [75]. EPA and DHA maintain the integrity of the intestinal barrier by reducing the permeability-induced increases in the levels of inflammatory cytokines, such as tumor necrosis factor *α* (TNF*α*), interferon *γ* (IFN*γ*), and IL-4. In addition, dietary omega-3 PUFAs, which affect intestinal integrity, have been shown to reduce clinical colitis and colonic immunopathology by improving epithelial barrier function in animal models [76]. In addition, as mentioned above, several lines of evidence support roles for both the microbiota and omega-3 PUFAs in the regulation of inflammation and the immune system. In particular, omega-3 PUFAs share an important immune system activation/inhibition pathway with gut microbes that modulate the profiles of proinflammatory factors [77].

### 4.3. The Host Immune-Microbiome Interaction Mediated by Omega-3 PUFAs

PUFAs simultaneously modulate the gut microbiota and immunity. Piglets fed omega-3 PUFA-enriched diets exhibit an increase in systemic and intestinal immunity, as evidenced by increased plasma concentrations of immunoglobulin G, decreased numbers of CD3+CD8+ T lymphocytes, and downregulated expression of intestinal genes (MyD88, NF-*κ*B, TNF-*α*, and IL-10). This diet also increased the amount of omega-3 PUFAs in the mucosa and decreased the ratio of omega-6/omega-3 PUFAs. In addition, the omega-3 PUFA-enriched diet decreased the abundance of pathogenic spirochaetes in the colonic digestive tract and increased the abundance of *Actinomycetes*, *Blautia* spp., and *Bifidobacteria* [78]. Omega-3 fatty acids inhibit the growth of gut microbiota associated with obesity and peptic ulcer disease and increase the proliferation of beneficial bacteria. The key to maintaining the steady state is a good ratio of omega-3 to omega-6 PUFAs, the former are anti-inflammatory molecules and the latter are proinflammatory molecules [79]. High saturated fat and omega-6 intake by stud rats induced alterations in the microbiota of their offspring, exacerbating inflammatory responses and conferring increased susceptibility to autoimmune, allergic, and infectious diseases [80]. Omega-3 PUFAs reduce the inflammatory response associated with metabolic endotoxemia, which has been shown to affect the gut microbiota, by promoting the growth of *Bifidobacteria* [51]. In addition, supplementation with omega-3 PUFAs relieves gut microbial dysbiosis caused by early life stress [50]. Based on these results, omega-3 PUFAs potentially alter gut immunity, which may be associated with altering the type and abundance of gut microbiota.

Omega-3 PUFAs also maintain host immunity by maintaining the balance between beneficial and harmful bacteria. A decrease in beneficial bacteria leads to a weakened intestinal resistance to harmful bacteria, resulting in a strong activation of proinflammatory signaling pathways, such as LPS-producing bacteria activate the NF-*κ*B signaling pathway by binding to TLR-4 on intestinal epithelial cells, which subsequently leads to the secretion of proinflammatory cytokines [81]. Various studies have found that omega-3 PUFAs can reverse gut microbial dysbiosis by increasing probiotic species (including *Lactobacillus* and *Bifidobacterium*) and butyric acid-producing bacteria [52].

Omega-3 PUFAs may modulate immune responses through several potential mechanisms. Primarily, omega-3 PUFAs directly modulate systemic immunity by altering the phospholipid membranes of immune cells, inhibiting omega-6-induced inflammation, downregulating inflammatory transcription factors, or serving as precursors of anti-inflammatory lipid mediators. The intestinal microbiota in the offspring of mice fed high-omega-3 diets was altered, with a moderate increase in the levels of the anti-inflammatory cytokine IL-10 in both the colon and spleen [82]. Increased levels of omega-3 PUFAs alter the phospholipid membrane composition of immune cells, thereby affecting proinflammatory signaling pathways. Alterations in regulatory T cell (Treg) function may be another potential explanation for omega-3 PUFA-mediated changes in host immunity and gut microbes. Omega-3 PUFAs prevent allergic diseases and reduce inflammatory responses by increasing the number of Treg cells. However, our findings from methicillin-resistant *Staphylococcus aureus* (*S. aureus*) skin infections are inconsistent with the findings from human studies suggesting a protective effect of enhanced Treg function on *S. aureus* infection [38]. Finally, current knowledge of how dietary fats alter the microbiome includes the TLR4-dependent induction of local inflammation that leads to alterations in the host environment, shifts in immune cell membrane function, and changes in nutrient availability that favor some organisms over others. Overall, these studies prompted researchers to propose associations among omega-3 PUFAs intake, alterations in the gut microbiome, and the regulation of the immune system, which may prevent associated inflammatory diseases [34].

## 5. Factors Associated with Omega-3 PUFA-Microbiome-Host Immunity Interactions

Many factors can affect omega-3 PUFA-microbiome-host immunity interactions, containing obesity, cancer, genetic disorders, and metabolic diseases. Omega-3 PUFAs may interfere with the development of obesity by modulating the gut microbiota and influencing the function of white adipose tissue [83]. Supplementation with omega-3 PUFAs can decrease blood lipids, and a meta-analysis included 2,630 showed that ALA significantly decreased triglycerides, LDL-cholesterol, and VLDL-cholesterol [84]. Evidence suggests that omega-3 PUFAs have anticancer activity, modulating cancer development by maintaining cell proliferation signals, inhibiting growth inhibitors and cell death, promoting angiogenesis, and reducing inflammation [85]. In genetic diseases such as epilepsy, although there are studies suggesting that omega-3 PUFAs may be beneficial, but the current research is insufficient to support this conclusion [86, 87].

### 5.1. Obesity

Obesity is associated with low-grade systemic inflammation. The consumption of a high-fat diet modulates the gut microbiota to substantially increase intestinal permeability, leading to LPS absorption and metabolic endotoxemia that triggers inflammation and metabolic disorders [88]. In particular, a high-fat diet is implicated in enteric dysbacteriosis, including a decrease in the abundance of Bacteroidetes, an increase in the abundance of both Firmicutes and Proteobacteria in the murine model, a reduction in the microbiota richness in terms of the number of species per sample, an increase in the abundance of LPS-producing bacteria such as Enterobactericeae, and/or a decrease in the abundance LPS-suppressing bacteria (species that decrease the numbers of LPS-producing bacteria, such as *Bifidobacterium*).

Obese patients exhibit impair intestinal immunity due to a reduced gut microbial diversity, and metabolic pathway alteration leads to the level of DHA and EPA decreased, which can be alleviated by supplementation with omega-3 PUFAs [89]. In addition, obese patients usually present low levels of inflammation, which is often associated with metabolic syndrome. Oral administration of omega-3 PUFAs alleviates inflammation in fat mice, thereby enhancing the function of the immune system [90]. As the role of omega-3 PUFAs in treating obesity, preserving gut microbial diversity, and maintaining gut health, this may provide us a possible new approach to improve obesity by modulating omega-3 PUFAs, gut microbes, and gut health.

### 5.2. Nonalcoholic Fatty Liver Disease (NAFLD)

**Figure 3:**
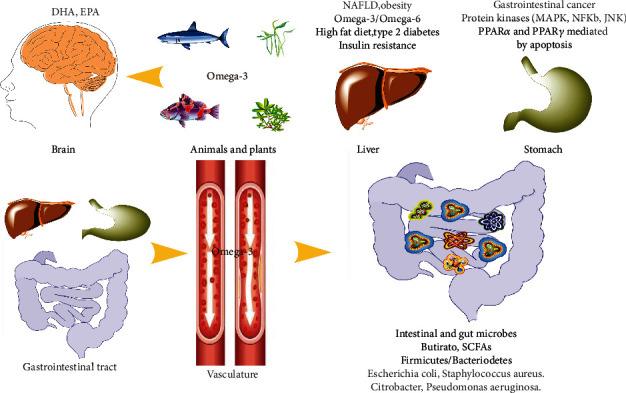
Factors associated with omega-3 PUFAs-microbiome-host immunity interactions.

Increased lipogenesis, hyperlipidemia, and increased fat deposition contribute to NAFLD development. NAFLD is characterized by triacylglycerol accumulation in hepatocytes (steatosis), which may progress to inflammation, fibrosis, and cirrhosis (steatohepatitis). Numerous studies have implicated the gut microbiota in the development of NAFLD (Figure 3), as it specifically mediates the interaction between nutrient intake and gut-liver function. The administration of *Lactobacillus rhamnosus* to NAFLD mice for 8 weeks increases the abundance of beneficial bacteria in the distal small intestine and decreases portal alanine aminotransferase activity, thereby reducing the symptoms of NAFLD [91].

Meanwhile, omega-3 and omega-6 PUFAs (omega-3/omega-6 PUFAs) have been linked to NAFLD [75, 92]. The omega-3/omega-6 balance is important for maintaining human health. In recent years, the percentage of omega-6 PUFAs in Western diets has increased significantly, disrupting this balance and increasing the incidence of various inflammatory diseases, such as obesity, NAFLD, and insulin resistance [93]. Currently, many clinical studies have reported that supplementation with fish oil, seal oil, and purified omega-3 PUFAs can reduce hepatic lipid content in individuals with NAFLD. Hepatic steatosis is alleviated by omega-3 PUFAs in individuals with NAFLD. In patients with NAFLD, administration of high concentrations of omega-3 significantly increased the omega-3 index and absolute values of EPA and DHA in red blood cells (RBC) and reduced the RBC omega-6/omega-3 fatty acid ratio (*P* < 0.0001) [94]. In rats fed a high-fat diet, combined omega-3 PUFA supplementation protected the animals from the development of severe NAFLD [95].

### 5.3. Gastrointestinal Malignancies or Cancer

Omega-3 PUFAs are important lipids that participate in many pathological processes related to tumor occurrence and development by relieving inflammation [4]. Omega-3 PUFAs may protect against cancers, including colorectal, breast, and prostate cancer (Figure 3). A study of 68,109 Washington residents found that omega-3 PUFAs reduced the risk of colon cancer in men, but had no significant effect on women or on rectal cancer [96]. Another meta-analysis showed an inverse relationship between EPA and DHA levels and colorectal cancer [97]. However, the relationship between omega-3 PUFA intake and colorectal cancer remains controversial, as a meta-analysis of 8,875 patients showed that omega-3 PUFAs tended to reduce the risk of cancer in the proximal colon but increased the risk of distal colon cancer [98]. Studies using a mouse model have shown that EPA supplementation decreases the number and size of tumors and increases body weight, changes that are associated with inhibition of COX-2 and reduced *β*-catenin nuclear translocation [99]. While omega-3 PUFAs inhibit tumor growth and relieve inflammation, they do not prevent the damage caused by cancer.

One possible explanation is that the modulation of the intestinal microbiota may contribute to the cancer-preventative properties of omega-3 PUFAs. Free feeding of EPA on mice with colon cancer increased the abundance of lactic acid-producing bacterial species in the gut [100]. Patients with colorectal cancer exhibit significant intestinal dysbiosis, including reduced microbial diversity and richness and impaired intestinal immunity [101]. In patients with severe cases, symptoms such as diarrhea, intestinal bleeding, and localized ulceration may occur, weakening the immune system. Colorectal cancer significantly decreases immunity and gut microbial diversity, and although omega-3 PUFAs reduce inflammation, the effect is not significant.

### 5.4. Bacterial and Viral Infections

Omega-3 PUFAs may play a key role in the host defense against infections by limiting excess inflammation and enhancing the immune response [35], but bacterial and viral infections compromise the effectiveness of omega-3 PUFAs. Omega-3 PUFA-enriched diets promote the colonization of beneficial bacteria and protect against the growth of pathogenic bacteria [62], thereby maintaining gut microbes in a healthy physiological environment and enhancing gut immunity. *Staphylococcus aureus* produces enterotoxins in the human intestinal tract that wreak havoc on the human gut, causing symptoms such as vomiting and diarrhea [102]. Omega-3 PUFAs inhibit *Staphylococcus aureus*, and DHA and EPA have been used clinically as topical agents to treat skin lesions caused by *Staphylococcus aureus* [102]. *Citrobacter* is a bacterium present in the intestinal tract of mice that promotes the proliferation of other pathogenic bacteria in the intestinal tract and causes gastrointestinal disease. An experiment conducted using mice with colitis showed that the administration of omega-3 PUFA-rich diet for 3 weeks altered the phospholipid composition of the intestinal cell membrane, reduced local inflammation, and reduced the production of proinflammatory cytokines and chemokines, thereby reducing colonic damage [103]. The consumption of omega-3 PUFA-rich foods for 5 weeks affected the intestinal microbiota, reducing the amount of *Clostridium perfringens* (a bacterium associated with IBD) and increasing the amount of *Lactobacillus* spp. and *Bifidobacterium spp*. with anti-inflammatory properties [104, 105]. The intake of 500 mg/d omega-3 PUFAs by adults reduced infections caused by *Escherichia coli*, *Staphylococcus aureus*, *Pseudomonas aeruginosa*, and *Streptococcus pneumoniae* and reduced the incidence of pneumococcal infections in the elderly [35]. The species and functions of gut microbes are complex, and a large number of microbes that are potentially influenced by omega-3 PUFAs will be gradually identified.

## 6. Summary and Perspectives

We reviewed the interactions among PUFAs, gut microbes, and host immunity. Based on accumulating evidence, omega-3 PUFAs (DHA, EPA, and ALA) exert profound effects on the intestinal microbiota, the host-microbiome interaction, and interactions between the host immune system and gut microbiota. Accordingly, the gut microbiota modulates the absorption and metabolism of omega-3 PUFAs and directly or indirectly modulates subsequent physiological and immune responses in the host. In previous studies, researchers focused on the trends in the host digestion and absorption of omega-3 PUFAs, while the effects of gut microbes on omega-3 PUFAs have often been neglected. Therefore, further comprehensive studies about the effects of omega-3 PUFAs on gut microbes and gut immunity will be meaningful. Likewise, we also must determine which gut microbes, which type of omega-3 PUFAs, or which pathways affect gut microbial homeostasis and host immunity.

Factors such as obesity and diseases are associated with host gut microbes, gut immunity, and omega-3 PUFAs. Omega-3 PUFAs modulate gut immunity by acting on gut microbes. In addition, omega-3 PUFAs are a feasible approach to maintain gut health. However, the composition of the gut microbes is complex, and simply using one substance will not be an effective method to solve these problems; individualized treatments for patients should be developed.
